# A Mini Review on Yolk-Shell Structured Nanocatalysts

**DOI:** 10.3389/fchem.2020.606044

**Published:** 2020-11-30

**Authors:** Xiaohuan Sun, Jie Han, Rong Guo

**Affiliations:** School of Chemistry and Chemical Engineering, Yangzhou University, Yangzhou, China

**Keywords:** yolk-shell, nanocatalyst, size selective, tandem catalysis, yolk-in-shell

## Abstract

Yolk-shell structured nanomaterials, possessing a hollow shell and interior core, are emerging as unique nanomaterials with applications ranging from material science, biology, and chemistry. In particular, the scaffold yolk-shell structure shows great promise as a nanocatalyst. Specifically, the hollow shell offers a confined space, which keeps the active yolk from aggregation and deactivation. The inner void ensures the pathway for mass transfer. Over the last few decades, many strategies have been developed to endow yolk-shell based nanomaterials with superior catalytic performance. This minireview describes synthetic methods for the preparation of various yolk-shell nanomaterials. It discusses strategies to improve the performance of yolk-shell catalysts with examples for engineering the shell, yolk, void, and related synergistic effects. Finally, it considers the challenges and prospects for yolk-shell nanocatalysts.

## Introduction

Core-shell nanomaterials have received enormous attention owing to their unique structure related properties and the broad variety of ways in which they can be applied in energy storage (Xie et al., 2015; Lu et al., 2019), sensing (Gong et al., 2019), cancer therapy (Wang et al., 2018b; He et al., 2020), and in particular, catalysis (Gawande et al., 2015; Das et al., 2020; Salvatore et al., 2020). By tuning the intrinsic properties of the core or shell, a variety of nanocatalysts, with active centers that are properly shielded by permeable shells, are ready to be designed and fabricated (Murugesan et al., 2020; Wang et al., 2020). Noveron et al. originally developed a green and sustainable methodology with metal nanoparticles encapsulated in tea leaves (Ahsan et al., 2020a) or tissue papers (Ahsan et al., 2020b,c). After carbonization, core-shell nanocatalysts with excellent electrocatalytic performance were prepared. In comparison with the other nanocatalyst systems, such as metallic nanoparticles or surface coating hybrids, the creation of a core-shell nanostructure significantly improved instability by keeping the active center from coalescence (Zhang et al., 2013). Though the stability issue was greatly enhanced, the surface area of the catalytic core was largely blocked by the shell, therefore, there is still space for the development of nanocatalysts with superior performance.

The yolk-shell nanostructure, which as an exterior hollow shell and interior movable core, has received increasing attention since it was first reported (Yin et al., 2004). Compared with core-shell, yolk-shell has an extra void space with an excellent catalytic performance in several ways. Among these benefits, the yolk-shell scaffolds can provide a field of catalysis, with three major aspects that should be highlighted: (1) the total exposure of the active center inside the shell is to some degree balanced by the contradiction of catalytic efficiency and stability; (2) the presence of void largely expands the space for the occurrence of catalytic reaction and mass transfer; (3) the manipulation of shell, yolk, void, or a combination of these enables the flexible and dynamic modulation of the catalytic efficiency, stability, recyclability or even synergistic effect induced multifunction. In this minireview, we briefly introduce synthetic methods for creating yolk-shell nanomaterials. We then discuss recent strategies for optimizing the catalytic performance of a yolk-shell nanocatalyst and explore the structure-property relationship.

## Methods for the Preparation of Yolk-Shell Nanomaterials

Through the flexible manipulation of basic physiochemical principles, several methods have been developed to prepare yolk-shell nanomaterials. These include hard template, soft template, swelling and shrinkage, swelling and evaporation, ship-in-a-bottle, hydrothermal and solvothermal methods, among others, which can be categorized as the template-assisted and template-free methods (Li et al., 2019a). Taking advantage of alternative methods, yolk-shell nanomaterials with various features, which boosted the field of catalysis in different perspectives, were synthesized. In this section, we introduce the typical methods that allow the design, preparation, and modulation of the yolk-shell nanomaterials. By summarizing the pros and cons that each method owns, we anticipate providing an instructive guide for the desired preparation of yolk-shell nanocatalysts.

## Template-Assisted Methods

**Figure 1:**
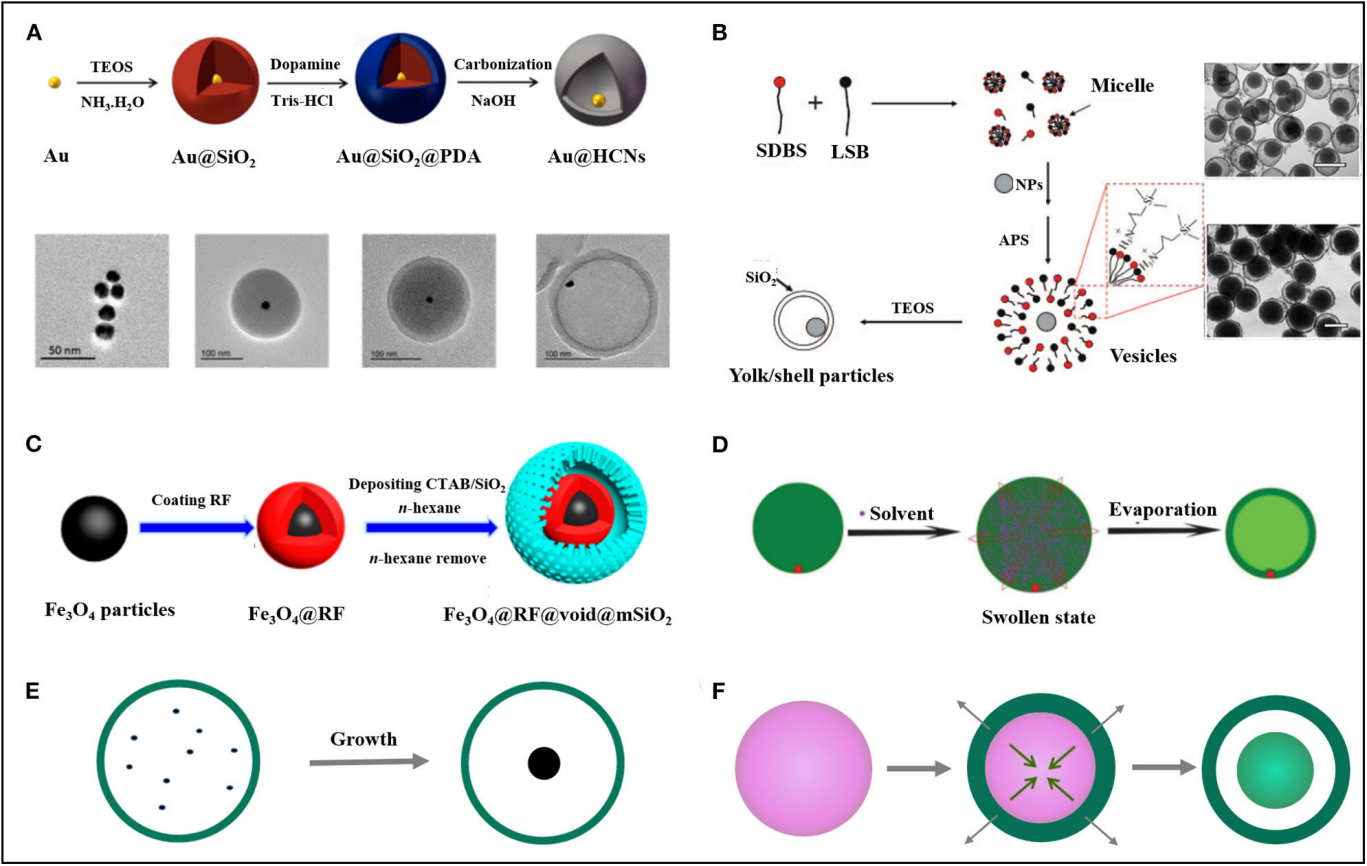
Illustration of the methods for the preparation of yolk-shell nanomaterials. **(A)** Hard-template method. Reprinted with permission from Yin et al. (2004). Copyright 2018 American Chemical Society. **(B)** Soft-template method. Reprinted with permission from Lee et al. (2008). Copyright 2009 American Chemical Society. **(C)** Plasmolysis inspired method. Reprinted with permission from Wu and Xu (2009). Copyright 2017 American Chemical Society. **(D)** Swelling-evaporation method. Reprinted with permission from Yu et al. (2019). Copyright 2014 Royal Society of Chemistry. **(E)** Ship in a bottle method. **(F)** Hydrothermal/solvothermal method. TEOS, tetraethyl orthosilicate; SDBS, sodium dodecyl benzenesulfonate; LSB, lauryl sulfonate betaine; RF, resorcinol-formaldehyde; CTAB, cetyltrimethylammonium bromide.

Template-assisted methods are usually performed according to the mechanism of layer by layer assembly and, due to the simplicity of this synthetic strategy, are widely employed in the preparation of yolk-shell nanostructures (Liu et al., 2014). Among the versatile template-assisted methods that have been developed to date, the hard template method is one of the most commonly adopted approaches. Typically, the method primarily involves synthesizing core-shell structures with a sacrifice inner shell. After the removal of the sacrifice layer, hollow spheres with an interior core are obtained. Fan et al. (2018) developed the standard procedure of yolk-shell fabrication, taking advantage of this method (Figure 1A). In this example, pre-prepared AuNPs were capped by SiO_2_ shells through the hydrolysis of tetraethyl orthosilicate (TEOS), resulting in Au@SiO_2_. Subsequently, polymerization of dopamine occurred on the surface of the Au@SiO_2_, after which Au@SiO_2_@polydopamine was obtained. Then, the materials were carbonized to convert the polymer shell into a carbon sphere. In the end, the SiO_2_ layer was etched with NaOH and Au@C yolk-shell structured nanomaterials were prepared. In this method, SiO_2_ was employed as a sacrifice layer due to its low cost and the simplicity with which it can be removed. Carbon sphere (Zhang et al., 2015) and polymers (Joo et al., 2016) are also commonly used as a sacrifice layer. To avoid the preparation of an extra template shell, selectively etching of the shell (Liang et al., 2017) or core (Lee et al., 2008) of a core-shell nanostructure provided alternative ways to fabricate yolk-shells with a tailored interior cavity and a controllable size of the active core.

Although yolk-shell nanomaterials can be elaborately designed and synthesized, the hard template method exhibits several disadvantages, such as the tedious nature of the procedure, the harsh conditions for template etching, a necessity for surface functionalization, and the fact that it is also time-consuming (Fan et al., 2012; Guiet et al., 2015). To circumvent the above-mentioned problems, the soft template method was developed. In this method, a mixture of surfactants, which can easily form micelles or vesicles, were employed to encapsulate core materials. Then, with the replication of the morphology formed by the surfactant, a hollow sphere encapsulated with the desired core can be synthesized in a relatively simple way. Wu et al. have presented a method for preparing the yolk/silica materials, taking advantage of the soft template strategy (Figure 1B) (Wu and Xu, 2009). In their strategy, core materials were first dispersed in a mixed solution consisting of zwitterionic (lauryl sulfonate betaine, LSB) and anionic surfactant (sodium dodecyl benzenesulfonate, SDBS). Then, with the addition of 3-aminopropyltriethoxysilane (APS), the formation of vesicles with a movable core was promoted. Besides being a vesicle-inducing agent, APS can also interact with the surface of the vesicle through electrostatic attraction, acting as the costructure-directing agent. The hydrolysis of APS and TEOS led to the direct formation of a hollow SiO_2_ shell along the surface of the vesicle. Interestingly, the shells formed were adaptive to the size and shape of the core through self-adjustment. The template can be easily removed by washing or calcination depending on the intrinsic nature of the shell, core, and template. This methodology provides a general principle for the relatively simple preparation of various yolk-shell nanomaterials. This method is particularly productive since, by repeating the soft template driven process, yolk-multishell nanomaterials can also be designed (Wu and Xu, 2010).

## Template-Free Methods

Even though template-assisted methods are a productive approach for the preparation of yolk-shells, their application is largely limited due to the multistep reaction and consumption of the template (Yu et al., 2019). Thus, template-free methods with a simpler procedure have stimulated intensive attention. Various strategies have been developed based on the above consideration and the related synthetic mechanisms were focused on an external trigger induced volume variation, Ostwald ripening, or Kirkendall effect.

A template-free method inspired by the cell plasmolysis phenomena in nature was developed for yolk-shell preparation by Yue et al. (Figure 1C) (Yue et al., 2017). The mechanism of this strategy lies in the fact that the low cross-linking resorcinol-formaldehyde resin with loose morphology can undergo volume swelling and shrinkage upon being soaked in or extracted from a specific organic solvent. The strategy developed by Yue et al. primarily synthesized Fe_3_O_4_ NPs coated with resorcinol-formaldehyde (RF). Then, an SiO_2_ shell was deposited on the swollen surface of Fe_3_O_4_@RF in a mixed solvent of n-hexane/H_2_O. After the extraction of the solvent by the above system, the Fe_3_O_4_@RF sphere shrank and spherical Fe_3_O_4_@RF@void@mSiO_2_ yolk-shell nanostructures with uniform dispersion were obtained. It is worth noting that yolk-shell structures achieved in this way can reversibly transit between the compact core-shell and spacious yolk-shell by repeating the swelling and shrinkage process, which offer a unique flexibility of the as-prepared nanomaterial. Another example presented by Niu et al. also (Niu et al., 2019) demonstrated the feasibility of this principle. In their case, the yolk-shell nanostructures were fabricated via the modulation of the repulsive interaction between polystyrene-b-poly(acrylic acid) (PS-b-PAA) and CTAB by solvent extraction, which further directed their morphology transformation, leading to the cell plasmolysis like behavior induced yolk-shells formation.

Controllable tuning of the relative position between the exterior shell and interior yolk can modify the yolk-shell interaction. Meanwhile, the migration of the yolk close to the shell will enhance its contact probability with the external substrate, which represents a significant improvement in catalytic efficiency. Taking this into account, our group reported a yolk-in-shell synthetic strategy with the swelling-evaporation method (Figure 1D) (Han et al., 2007, 2014). In our method, asymmetric Au-poly(*o*-methoxyaniline) (POMA) core-shell was prepared with the proper modulation of the addition point of Au NP during the polymerization of *o*-methoxyaniline (OMA). After dispersing the Au@POMA core-shell into ethanol, the POMA shell underwent volume expansion due to the entering of the solvent. When ethanol was eventually evaporated, the polymer chain of POMA together with ethanol moved outward, enabling the transformation of the solid core-shell into a hollow sphere with a single AuNP embedded in the shell.

The starting point for the design and preparation of yolk-shell nanostructures is to create a confined microenvironment where the core material can maintain both its activity and stability. For the above mentioned synthetic methods, each displayed unique advantages, however, they all are limited by the fact that the core material has to be prepared in advance and, unavoidably, suffers from the harsh conditions during processing, such as high temperature and highly acidic or alkaline environments, which may have an adverse effect on the physiochemical properties of the core (Zhou et al., 2019). To address these problems, methods known as “ship in a bottle” (Xiao et al., 2012) have attracted recent attention. This approach involves preparing hollow shells in advance and seeding initiators of the core through diffusion. With the addition of appropriate reduction agents, the core material can grow in the preexisting hollow shell, without the risk of being exposed to the harsh conditions that are employed during the template removal (Figure 1E) (Goebl and Yin, 2013).

In the “ship in a bottle” method, the yolk material successfully avoided the harsh conditions for template removal, however, this synthetic procedure is a complex way of preparing the hollow sphere. The hydrothermal and solvothermal methods offer a one pot synthetic approach. In this approach, the precursors were dissolved in hot water (hydrothermal method) or organic solvent (solvothermal method) under high pressure in autoclave. In these conditions, the precursors grow into yolk-shell morphology with the mechanism of Ostwald ripening or Kirkendall effect (Figure 1F) (Wang et al., 2018a,c). Besides the simplified procedure, the formation of homo yolk-shell is also an advantage provided by this method. However, this method is only suitable for the fabrication of metal or metal oxide based yolk-shell architectures (Li et al., 2019a).

## Strategies to Optimize Yolk-Shell Material as Nanocatalyst

**Scheme 1:**
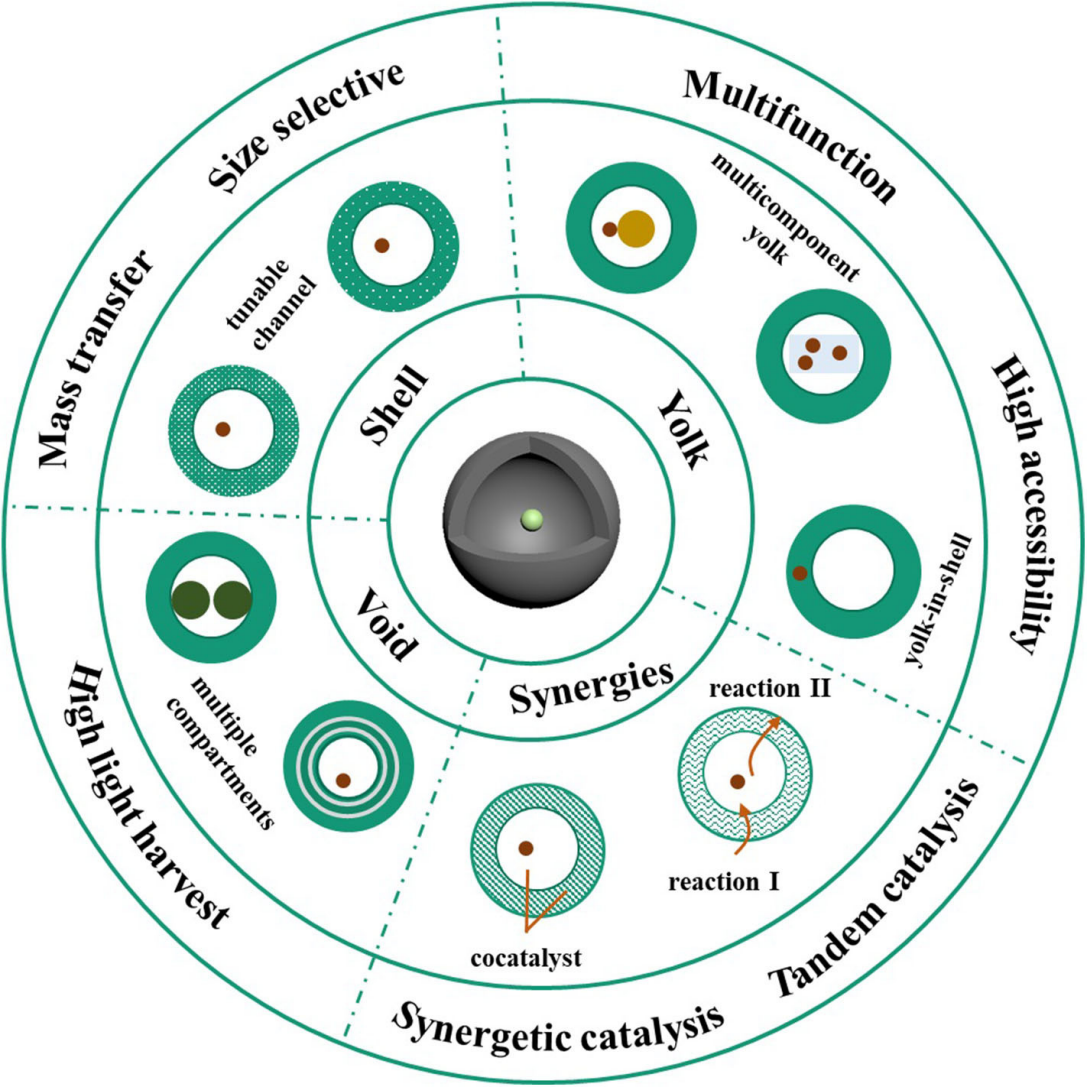
Schematic illustration of the structure related optimization of yolk-shell nanomaterials in catalysis.

Yolk-shell structured nanomaterials, with a unique hollow shell and interior core, have gained increasing attention in the field of catalysis due to the dual or multiple combination of components, which induce synergistic effect that provide the possibility for high performance catalysis. For the initial study of the yolk-shell scaffold as a catalyst, the shell was designed as a protecting agent to prevent the active metal yolk from aggregation and deactivation. Yin et al. reported the preparation of Pt@CoO in 2004 and confirmed its activity in the ethylene hydrogenation reaction, which, to the best of our knowledge, is the first yolk-shell based nanocatalyst (Yin et al., 2004). Afterwards, alternative yolk-shell materials were elaborately designed by tuning the categories of yolk and shell components. Lee et al. fabricated the Au@SiO_2_ yolk-shell nanomaterials, since Au is catalytically active and SiO_2_ shell is mesoporous and can be easily deposited on the nanosurface (Lee et al., 2008). Catalytic investigations have shown that the Au@SiO_2_ exhibits high efficiency toward the reduction of *p*-nitrophenol. Although modulation of the shell and active center significantly enhanced both the stability and efficiency of the as-prepared catalysts, the construction of superior catalysts is still challenging. In recent decades, novel methods have used flexible modulation of the shell, yolk, and void or a combination of them, to maximize catalytic efficiency. This section discusses the strategies employed to improve the performance of yolk-shell based materials as a robust catalyst (Scheme 1).

## Modulation of Shell

The shell layer of the yolk-shell catalyst plays an important role in the catalytic process. Even though the shell provides a physical barrier that keeps the active cores from aggregation, it also in some ways hinders the entering of the reactants into the existing products. Therefore, the appropriate modulation of the porosity of the shell can increase the contact probability between substrates and the active center. With a more elaborate design, the shells with specific pore range can offer the yolk-shell structured catalysts intrinsic selectivity (Hofer et al., 2018; Das et al., 2020). Jia et al. (2015) developed a method of preparing Au NPs, which are encapsulated in a hollow styrene shell, in which the size of the pores embedded is <0.8 nm. To investigate the size selective permeation of the yolk-shell, they studied the chemical transformation of benzaldehyde and its sterically crowded analog, 3, 5-di-tert-butyl benzaldehyde. The results showed that the conversion ratio for benzaldehyde is more than 70%, while the conversion ratio for 3, 5-di-tert-butyl benzaldehyde is <10%. Additionally, when cross-linked polymers were employed as the hollow shell, the pore size can be adjusted by solvents with various polarities due to the differential in swelling degree (Xu et al., 2019), which also leads to size selective catalysis.

## Modulation of Yolk

The interior yolk of a yolk-shell nanostructure acts as an active center where the reagents can undergo a chemical transformation. In principle, looking for highly efficient catalysts and embedding them into a well-designed hollow shell could be a feasible way to improve the catalytic performance of yolk-shell nanomaterial. Meanwhile, in terms of addressing catalyst limitations, there are other promising approaches such as the design of dual or multiple functional yolks. Our group synthesized the yolk-shell with dumbbell-like Pt-Fe_3_O_4_ encapsulated in the N-doped carbon hollow nanospheres (Sun et al., 2019). With the investigation of the reduction of 4-nitrophenol and oxidation of β-ionone to the epoxide, the as-designed yolk-shell exhibits highly improved catalytic efficiency due to the presence of the heterojunction interface, which largely benefits electron transfer. Moreover, the catalyst can be easily recycled by exploiting the magnetic field thanks to the intrinsic magnetic property of Fe_3_O_4_. Further studies have shown that with six successive cycles, the catalyst maintained its catalytic performance, indicating the high stability of the yolk-shell based catalyst.

It is well known that catalysts with a smaller size exhibit superior performance. However, most conventional yolk-shells are composed of a single core with a relatively large diameter, to prevent unwanted aggregation. Chen et al. reported a facile method for the preparation of Co@C-N yolk-shell, featured with Co cores embedded in N-doped carbon (C-N) nanosheet and a porous C-N shell (Chen et al., 2018). In this novel scaffold, the nanosheet encapsulated inside the hollow shell can anchor multiple tiny Co NPs, playing an important role in the enhancement of the catalytic efficiency and simultaneously preventing the deactivation of the catalytic centers. Owing to the multi-core and porous shell characteristics, the hollow Co@C-N nanoreactors were highly efficient in the aerobic oxidation of alcohols in neat water under atmospheric pressure in air and base-free conditions. Moreover, due to the intrinsic magnetic nature of Co NPs, the Co@C-N catalyst can be easily recycled by a magnet without significant loss of catalytic activity.

Although the yolk-shell scaffolds already render the active yolk high accessibility to the substrates, catalytic efficiency is still limited due to the relatively large distance between the substrates and the catalytic center. Taking the abovementioned problem into consideration, a feasible way to improve the catalytic performance of the yolk-shell nanocatalyst is to migrate the movable interior yolk into the shell. With this in mind, our group fabricated the Au-POMA yolk-in-shell nanomaterials by elaborately modulating the addition time of AuNP to the polymerization mixture of OMA (Han et al., 2014). Catalytic experiments demonstrated that in the reduction reaction of 4-nitrophenol to 4-aminophenol, the catalytic efficiency of Au-POMA yolk-in-shell is two times higher than that of the Au-POMA core-shell. The comparison between yolk-shell and yolk-in-shell catalysts was also investigated (Hu et al., 2020). The Au@C yolk-shell and Au@C yolk-in-shell were prepared with a single Au nanoparticle located inside the hollow cavity and encapsulated in the shell, respectively. The Au@C yolk-in-shell revealed much higher catalytic efficiency toward the 4-NP catalytic reduction and β-ionone catalytic oxidation compared to the Au@C yolk-shell. Interestingly, the relationship between the thickness of the shell and size of Au NP has a significant effect on the catalytic performance of the Au@C yolk-in-shell. The above examples illustrate how both the decoration and migration of yolk material can endow the yolk-shell nanocatalysts with a better performance.

## Modulation of Void

The existence of a void in the unique structure of the yolk-shell material is vital and functions in several ways, such as by providing an isolated space for confined catalysis, offering a high surface to volume ratio, promoting the reflection and scattering of light, and enhancing light absorbance efficiency and therefore, improving photocatalytic performance (Ren and Yu, 2019; Xiao et al., 2019). Considering the abovementioned feature of voids, the creation of multi-level voids has emerged as an efficient approach to optimizing the performance of yolk-shell catalysts. Liu et al. prepared a ZnFe_2_O4 double shelled material with a facile solvothermal method (Liu et al., 2017). The shells were mesoporous and the double-shell ZnFe_2_O_4_ was confirmed to possess the surface area of 126.7 m^2^ g^−1^. Due to the high surface area and scattering enhancement induced strong absorption of visible light, the kinetic constant of double-shell ZnFe_2_O_4_ toward the photocatalytic degradation of gaseous *o*-DCB is 1.46 and 1.82 times as high as that of yolk-shelled spheres and solid spheres. Another typical example method of creating multi-level voids is reported by Wang et al. (2012), who prepared a unique ZnO hollow sphere with double-yolks. Compared with the double-shelled architecture, the nanomaterials with double yolk-shelled morphology provide three interior compartments, which may significantly enhance light reflection and scattering in light harvesting. This was confirmed by photocurrent measurements. The photocatalytic ability was investigated by examining the degradation of Rhodamine B. The highest activity was achieved by the double yolk-shelled ZnO, compared with the single yolk-shelled ZnO and single-shelled hollow spheres of ZnO.

## Modulation of Yolk@void@shell Synergetic Effect

Besides the engineering of one component, another common and efficient approach to improving the activity of the yolk-shell catalyst is to enhance the synergistic effect between the shell and yolk. One of the typical examples is Au@TiO_2_ yolk-shell material. Li et al. reported the Gold Nanoparticle@TiO_2_ and Gold Nanorod@TiO_2_ yolk-shell nanostructures for photocatalytic reaction (Li et al., 2015). The design of this scaffold is based on the fact that TiO_2_ shows low activity in solar light and the combination of it with the plasmonic metal would significantly expand the solar light absorption range and favor visible light catalysis. As expected, the Au@TiO_2_ yolk-shell shows higher efficiency for the oxidation of benzyl alcohol. An examination of this mechanism showed that when irradiated with light, the electron generated from Au migrated to the conduction band of TiO_2_, leaving positive charges on the Au core. The generation of charge separation caused by the synergistic function of Au and TiO_2_ greatly benefits the oxidation reaction upon visible irradiation. To further improve the photocatalytic efficiency based on Au@TiO_2_ yolk-shell material, our group introduced graphene as the inner shell (Wang et al., 2015). In this case, graphene facilitates electron transfer from Au to TiO_2_, thus suppressing the recombination of the electron-hole pair. When used for H_2_ production under visible light, the H_2_ evolution rate of Au@r-GO/TiO_2_ hybrids is 1.8 times higher than that of Au@TiO_2_, confirming an enhanced synergistic effect.

The electrochemical water splitting reaction is a promising way of converting and storing renewable energy. With an elaborate design, this yolk-shell nanostructure provides several advantages in water splitting catalysis (Mei et al., 2018; Zhang et al., 2020). Tan et al. synthesized Au NP @ Ni(OH)_2_ nanomaterials and investigated their catalytic performance for oxygen-evolution reactions (OER) (Cai et al., 2020). They found that the introduction of Au core improved the OER activity of hollow Ni (OH)_2_. Further study indicated a strong electronic interaction between Au and Ni (OH)_2_ for Au NP @ Ni(OH)_2_ and the valence state of Ni (OH)_2_, which shifted to positive, indicating the production of a larger amount of active sites for OER. The above example illustrates that the synergistic effect of the yolk-shell nanostructure can be favorable for the electrocatalysis of water splitting reaction.

The synergistic effect provided by the unique structure of yolk-shell offered a promising application in the catalysis of tandem reaction. Liu et al. prepared Pd@Al-MSiO_2_ yolk-shell for the chemical transformation of glucose to 1, 2-propylene glycol (Lv et al., 2020). In this well-designed scaffold, the mesoporous SiO_2_ shell is doped with Al, which mainly exists in the form of 4-coordinate, provided the Lewis acid microenvironment and facilitated by the isomerization of glucose to fructose. The isomerization of glucose is the first rate-determining step for the conversion to 1, 2-propylene glycol. Then the resulting fructose underwent retro-aldol condensation in the voids and hydrogenation at the interface of the Pd nanoparticle. With this design, the undesired reaction routes were greatly suppressed. The catalytic transformations processed in the different compartment of yolk-shell make them excellent catalysts for performing tandem catalysis.

## Summary and Outlook

This minireview has summarized methods for the preparation of a yolk-shell nanocatalyst, illustrating the advantages and disadvantages of each method. We also discussed strategies that were recently adopted to improve the catalytic efficiency, stability, and recyclability in the regulation of shell, yolk, voids, and related synergistic effects. These examples demonstrate the promising potential application of yolk-shell structured nanomaterial as catalysts.

One pot synthetic methods, which are suitable for general materials, represent a promising way of further boosting this area of research in the future. Another direction lies in the minimization of the size of the active core, which will undoubtedly optimize the catalytic performance of yolk-shell nanocatalysts. Pickering microcapsule (Wang et al., 2018d) with proper encapsulated inner material, exhibit yolk-shell like structure and may provide a promising alternative for efficient catalysis due to their high interfacial activity (Shi et al., 2018; Li et al., 2019b). Moreover, more control over the tuning of the catalytic performance may bring new dynamic benefits. In terms of practical applications, the scaled-up preparation of yolk-shell catalysts deserves more attention. Additionally, the integration of low-cost and energy clean components could decrease the production price and facilitate the development of more sustainable options.

## Author Contributions

XS contributed to writing this review. JH and RG established the concept. JH supervised the review. All authors contributed to the article and approved the submitted version.

## Conflict of Interest

The authors declare that the research was conducted in the absence of any commercial or financial relationships that could be construed as a potential conflict of interest.

## References

[B1] Ahsan M. A. Imam M. A. Puente Santiago A. R. Rodriguez A. Alvarado-Tenorio B. Bernal R. (2020a). Spent tea leaves templated synthesis of highly active and durable cobalt-based trifunctional versatile electrocatalysts for hydrogen and oxygen evolution and oxygen reduction reactions. Green Chem. 22, 6967–6980. 10.1039/D0GC02155E

[B2] Ahsan M. A. Puente Santiago A. R. Hong Y. Zhang N. Cano M. Rodriguez-Castellon E.. (2020b). Tuning of trifunctional NiCu bimetallic nanoparticles confined in a porous carbon network with surface composition and local structural distortions for the electrocatalytic oxygen reduction, oxygen and hydrogen evolution reactions. J. Am. Chem. Soc. 142, 14688–14701. 10.1021/jacs.0c06960 32786805

[B3] Ahsan M. A. Puente Santiago A. R. Sanad M. F. Mark Weller J. Fernandez-Delgado O. Barrera L. A.. (2020c). Tissue paper-derived porous carbon encapsulated transition metal nanoparticles as advanced non-precious catalysts: carbon-shell influence on the electrocatalytic behaviour. J. Colloid. Interface Sci. 581, 905–918. 10.1016/j.jcis.2020.08.012 32956910

[B4] Cai R. Jin H. Yang D. Lin K.-T. Chan K. Sun J. (2020). Generalized preparation of Au NP @ Ni(OH)_2_ yolk-shell NPs and their enhanced catalytic activity. Nano Energy 71:104542 10.1016/j.nanoen.2020.104542

[B5] Chen H. Shen K. Mao Q. Chen J. Li Y. (2018). Nanoreactor of MOF-derived Yolk–Shell Co@C–N: precisely controllable structure and enhanced catalytic activity. ACS Catal. 8, 1417–1426. 10.1021/acscatal.7b03270

[B6] Das S. Perez-Ramirez J. Gong J. Dewangan N. Hidajat K. Gates B. C.. (2020). Core-shell structured catalysts for thermocatalytic, photocatalytic, and electrocatalytic conversion of CO_2_. Chem. Soc. Rev. 49, 2937–3004. 10.1039/C9CS00713J 32407432

[B7] Fan C. M. Zhang L. F. Wang S. S. Wang D. H. Lu L. Q. Xu A. W. (2012). Novel CeO_2_ yolk-shell structures loaded with tiny Au nanoparticles for superior catalytic reduction of p-nitrophenol. Nanoscale 4, 6835–6840. 10.1039/c2nr31713c 23023220

[B8] Fan L. Xu X. Zhu C. Han J. Gao L. Xi J.. (2018). Tumor catalytic-photothermal therapy with yolk-shell Gold@Carbon nanozymes. ACS Appl. Mater. Interfaces 10, 4502–4511. 10.1021/acsami.7b17916 29341583

[B9] Gawande M. B. Goswami A. Asefa T. Guo H. Biradar A. V. Peng D. L.. (2015). Core-shell nanoparticles: synthesis and applications in catalysis and electrocatalysis. Chem. Soc. Rev. 44, 7540–7590. 10.1039/C5CS00343A 26288197

[B10] Goebl J. Yin Y. (2013). Ship in a Bottle: *in situ* confined growth of complex yolk-shell catalysts. ChemCatChem 5, 1287–1288. 10.1002/cctc.201300129

[B11] Gong Y. Wu X. Chen J. Li W. Han N. Zhang D. (2019). Enhanced gas-sensing performance of metal@ZnO core–shell nanoparticles towards ppb-ppm level benzene: the role of metal–ZnO hetero-interfaces. New J. Chem. 43, 2220–2230 10.1039/C8NJ04621B

[B12] Guiet A. Göbel C. Klingan K. Lublow M. Reier T. Vainio U. (2015). Hydrophobic nanoreactor soft-templating: a supramolecular spproach to Yolk@Shell materials. Adv. Funct. Mater. 25, 6228–6240. 10.1002/adfm.201502388

[B13] Han J. Song G. Guo R. (2007). Fabrication of polymer hollow nanospheres by a swelling–evaporation strategy. J. Polym. Sci. A 45, 2638–2645. 10.1002/pola.22023

[B14] Han J. Wang M. Chen R. Han N. Guo R. (2014). Beyond yolk-shell nanostructure: a single Au nanoparticle encapsulated in the porous shell of polymer hollow spheres with remarkably improved catalytic efficiency and recyclability. Chem. Commun. 50, 8295–8298. 10.1039/C4CC01532K 24777116

[B15] He X. Peng C. Qiang S. Xiong L. H. Zhao Z. Wang Z.. (2020). Less is more: silver-AIE core@shell nanoparticles for multimodality cancer imaging and synergistic therapy. Biomaterials 238:119834. 10.1016/j.biomaterials.2020.119834 32058870

[B16] Hofer C. J. Grass R. N. Schneider E. M. Hendriks L. Herzog A. F. Zeltner M.. (2018). Water dispersible surface-functionalized platinum/carbon nanorattles for size-selective catalysis. Chem. Sci. 9, 362–367. 10.1039/C7SC03785F 29629105 PMC5868313

[B17] Hu J. Li R. Han J. Sun J. Wang Y. Yu L.. (2020). Yolk–shell or yolk-in-shell nanocatalysts? A proof-of-concept study. J. Mater. Chem. A 8, 10217–10225. 10.1039/C9TA13390A26515784

[B18] Jia Y. Shmakov S. N. Register P. Pinkhassik E. (2015). Size-selective yolk-shell nanoreactors with nanometer-thin porous polymer shells. Chemistry 21, 12709–12714. 10.1002/chem.201501968 26223572

[B19] Joo J. B. Liu H. Lee Y. J. Dahl M. Yu H. Zaera F. (2016). Tailored synthesis of C@TiO2 yolk–shell nanostructures for highly efficient photocatalysis. Catal. Today 264, 261–269. 10.1016/j.cattod.2015.09.008

[B20] Lee J. Park J. C. Song H. (2008). A nanoreactor framework of a Au@SiO_2_ Yolk/Shell structure for catalytic reduction of *p*-nitrophenol. Adv. Mater. 20, 1523–1528. 10.1002/adma.200702338

[B21] Li A. Zhang P. Chang X. Cai W. Wang T. Gong J. (2015). Gold Nanorod@TiO_2_ Yolk-shell nanostructures for visible-light-driven photocatalytic oxidation of benzyl alcohol. Small 11, 1892–1899. 10.1002/smll.201403058 25641771

[B22] Li A. Zhu W. Li C. Wang T. Gong J. (2019a). Rational design of yolk-shell nanostructures for photocatalysis. Chem. Soc. Rev. 48, 1874–1907. 10.1039/C8CS00711J 30525133

[B23] Li Q. Zhao T. Li M. Li W. Yang B. Qin D. (2019b). One-step construction of Pickering emulsion via commercial TiO_2_ nanoparticles for photocatalytic dye degradation. Appl. Catal. B Environ. 249, 1–8. 10.1016/j.apcatb.2019.02.057

[B24] Liang Y. Yu K. Xie J. Zheng Q. Wang T. J. (2017). High hiding power and weather durability of film-coated titanium dioxide particles with a yolk-shell structure. Colloids Surf. A 520, 736–742. 10.1016/j.colsurfa.2017.02.046

[B25] Liu B. Li X. Zhao Q. Hou Y. Chen G. (2017). Self-templated formation of ZnFe_2_O_4_ double-shelled hollow microspheres for photocatalytic degradation of gaseous o-dichlorobenzene. J. Mater. Chem. A 5, 8909–8915. 10.1039/C7TA02048A

[B26] Liu R. Qu F. Guo Y. Yao N. Priestley R. D. (2014). Au@carbon yolk-shell nanostructures via one-step core-shell-shell template. Chem. Commun. 50, 478–480. 10.1039/C3CC47050D 24266025

[B27] Lu W. Guo X. Luo Y. Li Q. Zhu R. Pang H. (2019). Core-shell materials for advanced batteries. Chem. Eng. J. 355, 208–237. 10.1016/j.cej.2018.08.132

[B28] Lv M. Zhang Y. Xin Q. Yin D. Yu S. Liu S. (2020). Pd@Al-containing mesoporous silica Yolk–Shell-structured nanospheres as high performance nanoreactors for the selective hydrogenolysis of glucose to 1,2-propylene glycol. Chem. Eng. J. 396:125274 10.1016/j.cej.2020.125274

[B29] Mei G. Liang H. F. Wei B. B. Shi H. H. Ming F. W. Xu X. (2018). Bimetallic MnCo selenide yolk shell structures for efficient overall water splitting. Electrochim. Acta. 290, 82–89. 10.1016/j.electacta.2018.09.062

[B30] Murugesan K. Chandrashekhar V. Kreyenschulte C. Beller M. Rajenahally J. (2020). A general catalyst based on cobalt-core-shell nanoparticles for hydrogenation of N-Heteroarenes including pyridines. Angew. Chem. Int. Ed. 59, 17408–17412. 10.1002/anie.202004674 32543735 PMC7540604

[B31] Niu D. Jiang Y. He J. Jia X. Qin L. Hao J. (2019). Extraction-induced fabrication of yolk-shell-structured nanoparticles with deformable micellar cores and mesoporous silica shells for multidrug delivery. ACS Appl. Biol. Mater. 2, 5707–5716. 10.1021/acsabm.9b00759 35021564

[B32] Ren H. Yu R. (2019). Hollow multi-shelled structures for energy conversion and storage applications. Inorg. Chem. Front. 6, 2239–2259. 10.1039/C9QI00634F

[B33] Salvatore K. L. Deng K. Yue S. McGuire S. C. Rodriguez J. A. Wong S. S. (2020). Optimized microwave-based synthesis of thermally stable inverse catalytic core-shell motifs for CO_2_ hydrogenation. ACS Appl. Mater. Interfaces 12, 32591–32603. 10.1021/acsami.0c06430 32657113

[B34] Shi M. Yang R. Li Q. Lv K. Miron R. J. Sun J.. (2018). Inorganic self-assembled bioactive artificial proto-osteocells inducing bone regeneration. ACS Appl. Mater. Interfaces 10, 10718–10728. 10.1021/acsami.8b00385 29528210

[B35] Sun J. Hu J. Han J. Yuan G. Guo R. (2019). Dumbbell-like Pt-Fe_3_O_4_ Nanoparticles encapsulated in N-doped carbon hollow nanospheres as a novel Yolk@Shell nanostructure toward high-performance nanocatalysis. Langmuir 35, 12704–12710. 10.1021/acs.langmuir.9b02237 31490690

[B36] Wang A. Zhu Q. Xing Z. (2020). Multifunctional quaternized chitosan@surface plasmon resonance Ag/N-TiO_2_ core-shell microsphere for synergistic adsorption-photothermal catalysis degradation of low-temperature wastewater and bacteriostasis under visible light. Chem. Eng. J. 393:124781 10.1016/j.cej.2020.124781

[B37] Wang B. Yu Q. Zhang S. Wang T. Sun P. Chuai X. (2018a). Gas sensing with yolk-shell LaFeO3 microspheres prepared by facile hydrothermal synthesis. Sens. Actuat. B Chem. 258, 1215–1222. 10.1016/j.snb.2017.12.018

[B38] Wang C. Xu C. Xu L. Sun C. Yang D. Xu J.. (2018b). A novel core-shell structured upconversion nanorod as a multimodal bioimaging and photothermal ablation agent for cancer theranostics. J. Mater. Chem. B 6, 2597–2607. 10.1039/C7TB02842C 32254478

[B39] Wang J. Chen M. Yan X. Zhou C. Wang Q. Wang D. (2018c). A facile one-step hydrothermal synthesis of carbon–MoS2 yolk–shell hierarchical microspheres with excellent electrochemical cycling stability. J. Appl. Electrochem. 48, 509–518. 10.1007/s10800-018-1184-4

[B40] Wang M. Han J. Xiong H. Guo R. (2015). Yolk@Shell Nanoarchitecture of Au@r-GO/TiO_2_ hybrids as powerful visible light photocatalysts. Langmuir 31, 6220–6228. 10.1021/acs.langmuir.5b01099 25996904

[B41] Wang X. Chen L. Sun G. Liu R. (2018d). Hollow Microcapsules with controlled mechanical properties templated from Pickering emulsion droplets. Macromol. Chem. Phys. 220:1800395 10.1002/macp.201800395

[B42] Wang X. Liao M. Zhong Y. Zheng J. Y. Tian W. Zhai T.. (2012). ZnO hollow spheres with double-yolk egg structure for high-performance photocatalysts and photodetectors. Adv. Mater. 24, 3421–3425. 10.1002/adma.201201139 22674659

[B43] Wu X. Xu D. (2009). Formation of yolk SiO_2_ shell structures using surfactant mixtures as template. J. Am. Chem. Soc. 131, 2774–2775. 10.1021/ja808452r 19199444

[B44] Wu X. J. Xu D. (2010). Soft template synthesis of yolk/silica shell particles. Adv. Mater. 22, 1516–1520. 10.1002/adma.200903879 20437501

[B45] Xiao M. Wang Z. Lyu M. Luo B. Wang S. Liu G.. (2019). Hollow nanostructures for photocatalysis: advantages and challenges. Adv. Mater. 31:e1801369. 10.1002/adma.201801369 30125390

[B46] Xiao M. Zhao C. Chen H. Yang B. Wang J. (2012). “Ship-in-a-Bottle” growth of noble metal nanostructures. Adv. Funct. Mater. 22, 4526–4532. 10.1002/adfm.201200941

[B47] Xie Z. Ellis S. Xu W. Dye D. Zhao J. Wang Y. (2015). A novel preparation of core-shell electrode materials via evaporation-induced self-assembly of nanoparticles for advanced Li-ion batteries. Chem. Commun. 51, 15000–15003. 10.1039/C5CC05577F 26313024

[B48] Xu Y. Yao Y. Yu H. Shi B. Gao S. Zhang L. (2019). Nanoparticle-encapsulated hollow porous polymeric nanosphere frameworks as highly active and tunable size-selective catalysts. ACS Macro Lett. 8, 1263–1267. 10.1021/acsmacrolett.9b00490 35651158

[B49] Yin Y. Rioux R. Erdonmez C. Hughes S. Somorjai G. Alivisatos A. (2004). Formation of Hollow nanocrystals through the nanoscale kirkendall effect. Science 304, 711–714. 10.1126/science.1096566 15118156

[B50] Yu L. Pan P. Zhang Y. Zhang Y. Wan L. Cheng X.. (2019). Nonsacrificial self-template synthesis of colloidal magnetic yolk-shell mesoporous organosilicas for efficient oil/water interface catalysis. Small 15:e1805465. 10.1002/smll.201805465 30848869

[B51] Yue Q. Li J. Zhang Y. Cheng X. Chen X. Pan P.. (2017). Plasmolysis-inspired nanoengineering of functional yolk-shell microspheres with magnetic core and mesoporous silica shell. J. Am. Chem. Soc. 139, 15486–15493. 10.1021/jacs.7b09055 29016118

[B52] Zhang Q. Lee I. Joo J. Zaera F. Yin Y. (2013). Core shell nanostructured catalyst. Acc. Chem. Res. 46, 1816–1824. 10.1021/ar300230s 23268644

[B53] Zhang Q. Liu B. Ji Y. Chen L. Zhang L. Li L.. (2020). Construction of hierarchical yolk-shell nanospheres organized by ultrafine Janus subunits for efficient overall water splitting. Nanoscale 12, 2578–2586. 10.1039/C9NR08802D 31939458

[B54] Zhang W. Lin X. J. Sun Y. G. Bin D. S. Cao A. M. Wan L. J. (2015). Controlled formation of Metal@Al_2_O_3_ yolk-shell nanostructures with improved thermal stability. ACS Appl. Mater. Interfaces 7, 27031–27034. 10.1021/acsami.5b09791 26619036

[B55] Zhou M. Wang T. He Z. Xu Y. Yu W. Shi B. (2019). Synthesis of yolk–shell magnetic porous organic nanospheres for efficient removal of methylene blue from water. ACS Sustain. Chem. Eng. 7, 2924–2932. 10.1021/acssuschemeng.8b01807

